# From Clinical Symptoms to MR Imaging: Diagnostic Steps in Adenomyosis

**DOI:** 10.1155/2017/1514029

**Published:** 2017-12-04

**Authors:** H. Krentel, C. Cezar, S. Becker, A. Di Spiezio Sardo, V. Tanos, M. Wallwiener, R. L. De Wilde

**Affiliations:** ^1^Department of Gynecology, Obstetrics and Reproductive Health, Cayetano Heredia Peruvian University, Lima, Peru; ^2^Clinic of Gynecology, Obstetrics and Gynecological Oncology, University Hospital for Gynecology, Pius-Hospital Oldenburg, Medical Campus University of Oldenburg, Germany; ^3^Department of Obstetrics and Gynecology, Goethe University Frankfurt, Frankfurt, Germany; ^4^Department of Public Health, University of Naples “Federico II”, Naples, Italy; ^5^St. George's Medical School, Nicosia University, Aretaeio Hospital, Nicosia, Cyprus; ^6^Department of Obstetrics and Gynecology, University of Heidelberg, Heidelberg, Germany

## Abstract

Adenomyosis or endometriosis genitalis interna is a frequent benign disease of women in fertile age. It causes symptoms like bleeding disorders and dysmenorrhea and seems to have a negative effect on fertility. Adenomyosis can be part of a complex genital and extragenital endometriosis but also can be found as a solitary uterine disease. While peritoneal endometriosis can be easily diagnosed by laparoscopy with subsequent biopsy, the determination of adenomyosis is difficult. In the following literature review, the diagnostic methods clinical history and symptoms, gynecological examination, 2D and 3D transvaginal ultrasound, MRI, hysteroscopy, and laparoscopy will be discussed step by step in order to evaluate their predictive value in the diagnosis of adenomyosis.

## 1. Introduction

In the past, adenomyosis was diagnosed when histopathology revealed the disease after hysterectomy. Different publications report a rate of more than 30% of adenomyosis in hysterectomy specimens in premenopausal women undergoing hysterectomy for various indications [1]. However, the age depending incidence of adenomyosis, especially in young patients, so far remains unclear. Present evidence suggests that adenomyosis has a negative impact on female fertility [2, 3]. Salim et al. and Tremellen and Thalluri reported a decrease of pregnancy rates by 50% in women with adenomyosis undergoing IVF [4, 5]. Thalluri and Tremellen showed the negative impact on successful implantation following GnRH antagonists IVF treatment in patients with ultrasound diagnosed adenomyosis [6]. However, Benaglia et al. reported that asymptomatic adenomyosis diagnosed at transvaginal sonography does not impair implantation rates in IVF cycles [7]. Differences in study design, choice of patients and controls, and methods and parameters used to diagnose adenomyosis may explain these discrepancies. Kissler et al. concluded that in patients with intact tubo-ovarian anatomy adenomyosis might be the cause for subfertility [8]. Limited data from uncontrolled studies show that treatment of adenomyosis may improve infertility in women undergoing IVF [9]. Tao et al. showed that GnRH-antagonist cycles have an adverse effect on the outcome, while GnRH-agonist long cycle protocols may improve pregnancy rates and decrease abortion rates [10]. In combination with deep infiltrating endometriosis (DIE), the presence of adenomyosis also plays an important role. Vercellini et al. showed that adenomyosis was associated with a 68% reduction in the likelihood of pregnancy in women seeking conception after surgery for rectovaginal and colorectal endometriosis [11]. Lazzeri et al. reported that in 48.7% of patients with DIE also adenomyosis was found. The therapy of DIE reduced the symptoms. But in the group of patients with adenomyosis, the postsurgical result was significantly worse [12]. Thus, in the treatment of endometriosis related pain and/or infertility, it is of importance to know if adenomyosis is a possible cause of the symptoms. Screening for adenomyosis before suggesting surgical or medical treatment procedures may allow identification of subgroups and may lead to individual therapy planning [13]. But so far it remains difficult to diagnose adenomyosis as no reliable diagnostic standard exists. However, different diagnostic methods like clinical examination, transvaginal ultrasound, MRI, and hysteroscopy with guided biopsy have a high sensitivity and specificity in the hands of the skilled examiner. Especially with a standardized 2D-transvaginal ultrasound in combination with clinical symptoms and bimanual examination, considering the recently described sonographic parameters in adenomyosis has the potential to be a reliable, cost-effective, and accessible tool in the diagnosis of adenomyosis.

## 2. Methods

PubMed search has been conducted using the keywords adenomyosis, hysteroscopy, 2D transvaginal sonography, 3D transvaginal sonography, doppler sonography, elastography, MR imaging and laparoscopy.

## 3. Results and Discussion

The diagnosis of adenomyosis is difficult, especially in young premenopausal women. Different diagnostic methods offer subtle predictive factors in order to determine adenomyosis. Its combination in daily practice can help to ensure the diagnosis with or without a respective histopathologic proof (Table 1). Usually the clinical symptoms in combination with the result of the gynecological examination guide the way to the suspicion of adenomyosis [14]. In a next step, the transvaginal 2D and 3D sonography considering the typical signs of adenomyosis can confirm the clinical aspects and strengthen the diagnosis of adenomyosis [15]. In some cases, MR imaging can be helpful in order to determine the localization and size of the adenomyotic lesion and differentiate it from fibroids. Hysteroscopy and laparoscopy can then facilitate biopsies or surgical treatment options. In all mentioned steps, the accuracy of the diagnostic methods depends on the experience and skills of the examiner and requires a respective learning curve.

### 3.1. Clinical History and Symptoms

Adenomyosis is associated with dysmenorrhea, uterine bleeding disorders, chronic pelvic pain, and dyspareunia [1]. Li et al. reported that, in 710 premenopausal patients with adenomyosis, only 4.5% had no symptoms. With a rate of 81.7%, dysmenorrhea was the most common complaint [16]. Krentel described a rate of 60% of adenomyosis in uterine specimens after hysterectomy in a group of patients with the indication dysmenorrhea and bleeding disorders for surgery [17]. Thus, in patients with dysmenorrhea, the presence of adenomyosis should be assumed and determined by further diagnostic steps. However, clinical features in adenomyosis can change in relation to the patients age [18]. In young patients, the symptoms dysmenorrhea and chronic pelvic pain usually are correlated with the possibility of a peritoneal endometriosis and lead to diagnostic laparoscopy. Typical diagnostic imaging features of adenomyosis might be missing in such cases. However, persistent dysmenorrhea after complete laparoscopic resection of extrauterine endometriosis could be a sign for the presence of adenomyosis [19].

### 3.2. Gynecological Examination

The clinical examination alone cannot detect uterine adenomyosis. In some cases, the uterus might be larger than normal, but the alterations of the uterine tissue cannot be diagnosed without imaging techniques. However, the bimanual examination can help to estimate uterine or pelvic pain, pain localization, uterine size and mobility, adnexial masses, and the presence of deep infiltrating endometriosis in the retrocervical region like the rectovaginal septum. This is of importance as deep infiltrating endometriosis is correlated with adenomyosis in almost every second case [12]. Thus, the gynecological examination allows more detailed information about severity and complexity of the disease in the planning of medical or surgical treatment [20]. As a next step, clinical history and gynecological examination should be combined with transvaginal ultrasound considering the diagnostic sonographic signs of adenomyosis.

### 3.3. 2D and 3D Transvaginal Ultrasound

In the last years, transvaginal sonography (TVS) has been described as a diagnostic tool in adenomyosis with a range of sensitivity from 65 to 81% and of specificity from 65 to 100% [21]. Several 2D and 3D features in TVS associated with adenomyosis have been reported in various publications. Di Donato et al. described the nine following parameters as main criteria in the diagnosis of adenomyosis by TVS: heterogeneous myometrium, hyperechoic or hypoechoic linear striation in the myometrium, myometrial anechoic lacunae or cysts, subendometrial microcysts, asymmetrical myometrial thickening of the uterine wall, globally uterine enlargement, the so-called question mark sign, thickening of the junctional zone, and hyperechoic myometrial areas [41–43]. Graziano et al. summarized these parameters in a pictorial review concluding that TVS provides easily recognizable diagnostic signs enabling the diagnosis of adenomyosis by every gynecologist [22]. In a systematic review, Dartmouth concluded that the presence of myometrial cysts, linear myometrial striations, poor delineation of the JZ, and a heterogeneous myometrium raise the probability of the presence of adenomyosis, while anteroposterior uterine asymmetry is not a useful feature [23]. Kepkep et al. reported that subendometrial linear striations have the highest accuracy in the sonographic diagnosis of adenomyosis [44]. A diagnostic accuracy of 75% has been reported for the presence of the question mark sign by Di Donato et al. [41]. In 2006, Dueholm postulated that the diagnosis of adenomyosis is suggested by the presence of three or more of the above-mentioned signs [24]. In 2009, Meredith et al. reported a probability of adenomyosis with an abnormal transvaginal ultrasound of 66.2%, while the probability of adenomyosis with a normal transvaginal ultrasound was only 9.1% [45]. Pinzauti et al. showed a significant relationship between the number of 2D-TVS features of diffuse adenomyosis and VAS score for dysmenorrhea. In their observational study, diffuse adenomyosis was found in 34% of 18–30-year-old nulligravid women with regular menstrual cycle and without endometriosis. An asymmetrical myometrial thickening of the uterine walls was the most common TVS feature observed [46]. Dakhly et al. reported a sensitivity of 83.95% and a specificity of 60% of 2D-TVS in the diagnosis of adenomyosis in 292 patients with clinical suspicion of adenomyosis. In combination with hysteroscopic endomyometrial biopsy, the specificity increased to 89% [25]. Exacoustos et al. described the presence of myometrial cysts as the most specific and heterogeneous myometrium as the most sensitive feature in 2D-TVS [26]. Bazot et al. compared transvaginal ultrasound with magnetic resonance imaging and described a sensitivity of 76.4% and a specificity of 92.8% in the diagnosis of adenomyosis with TVS. Myometrial cyst was the most sensitive and specific parameter. In this study, no difference in accuracy was found between 2D-TVS and MRI, but sensitivity was lower with ultrasound in patients with additional uterine myomas [30]. Di Donato et al. reported a sensitivity of 92% and a specificity of 88% of 2D-TVS in a group of 50 patients scheduled for hysterectomy due to symptoms of endometriosis or adenomyosis [41].

Recent studies indicate that 3D-TVS might be superior to 2D-TVS in the diagnosis of adenomyosis [27]. Especially in the evaluation of the junctional zone, which is altered by adenomyosis, the 3D technique allows a more detailed assessment. In 2011, Exacoustos et al. showed a good diagnostic accuracy for adenomyosis by 3D-TVS of the coronal uterine section with evaluation and measurement of the junctional zone [26]. Luciano et al. demonstrated high diagnostic accuracy of 3D-TVS in detection of site and position of adenomyosis in the uterine wall by obtaining targeted biopsies after sonography. The most specific parameters in 3D-TVS were JZ (max) > 8 mm, myometrial asymmetry, and hypoechoic striation. Considering two of the mentioned features, the accuracy of diagnosis reached 90% [28]. Sharma et al. reported a rate of 86% of ill-defined junctional zone in 3D-TVS in patients with adenomyosis. Interestingly the feature central vascularity was found in 93% of adenomyosis lesions in additional Doppler sonography, while leiomyomas showed peripheral vascularity in 89% of the cases. Considering PI, RI, and Vmax, sensitivity was 95.6%, specificity was 93.4%, the PPV was 88.6%, and the NPV was 97.6% in the diagnosis of adenomyosis. Thus, they concluded that additional Doppler sonography can help to diagnose adenomyosis and distinguish it from myomas [29]. Another additional sonographic technique in the diagnosis of uterine tumors is sonoelastography that measures tissue strain and stiffness. In a prospective cohort study, Stoelinga et al. showed that myometrium, myomas, and adenomyosis had different elastographic characteristics and color patterns and thus the technique was able to discriminate the lesions. The agreement with MRI results was excellent [47]. Acar et al. reported an increase of the myometrial stiffness in adenomyosis compared to normal myometrium measured with shear wave elastography [48]. However, this method so far does not play an important role in the diagnosis of adenomyosis. In conclusion, the accuracy of transvaginal ultrasound in the diagnosis of adenomyosis is very variable depending on the selected examination criteria and the observer variation. In different studies, in accordance with the respective actual recommendations, adenomyosis was diagnosed by TVS in the presence of one to three or more sonographic features. In many studies, the imaging result was compared to histopathologic results after hysterectomy. Usually those patients are older than many of the possibly affected population and therefore likely to be more symptomatic with advanced severity of disease and sonographic and MRI features might be easier to detect than in younger patients. Thus, the study results regarding sensitivity and specificity cannot be easily compared. Considering four apparently similar studies, the reported sensitivity and specificity of 2D-TVS for the diagnosis of adenomyosis range from 87.1% to 57.4% and 97.5% to 60.1% [23]. A consensus statement on sonographic features of the uterus by the MUSA (Morphological Uterus Sonographic Assessment) group summarizes the parameters and the use of terminology in the sonographic description of adenomyosis and uterine myomas [49]. However, a reliable standardized transvaginal ultrasound scheme considering the most specific and sensitive parameters in recent literature should be established.

### 3.4. Magnetic Resonance Imaging (MRI)

MRI is a useful technique in the detection of adenomyosis and especially in the differentiation between adenomyosis and uterine myomatosis. In a prospective cohort observational study, Stamatopoulos et al. described a sensitivity of 46.1%, a specificity of 99.2%, and a positive predictive value (PPV) of 92.3% of MRI in the diagnosis of adenomyosis [31]. Typical MRI parameters in uteri with adenomyosis are the focal or diffuse thickening of the junctional zone, an area of low-signal-intensity in the myometrium, and high-signal-intensity spots in the T2-weighted resonance. Bazot el al. reported a sensitivity of 77.5%, a specificity of 92.5%, and a PPV of 83.8% in a prospective study with 120 patients. Junctional zone thickness (JZ (max)) > 12 mm, a JZ (max) to myometrial thickness ratio >40%, and the presence of a high-signal-intensity myometrial spot were the most specific factors, while JZ (max) was the most sensitive value [30]. Normal junctional zone thickness is considered to be <8 mm [32]. Novellas et al. reported a diagnostic accuracy in adenomyosis by MR imaging of 85%. Junctional zone thickness > 12 mm was the most important finding [33]. In daily practice, the diagnostic tool TVS is more accessible and cost-effective followed by MR imaging and in many cases the accuracy is similar or even higher in TVS, especially when combined with 3D and Doppler. In uncertain cases, MR imaging can be helpful to determine the diagnosis. Adenomyosis also can present as a circumscribed adenomyoma or adenomyotic cystic region or polyp [34]. In these cases, the MRI allows a detailed description of localization and size of the lesion, especially in the preoperative planification for surgical resection of focal or diffuse adenomyosis in infertility treatment. The appearance of adenomyosis in MR imaging also changes under hormonal treatment.

### 3.5. Hysteroscopy

Diagnostic hysteroscopy is a simple minimally invasive method in order to detect pathologies in the uterine cavity. It can easily be combined with laparoscopy and perturbation of fallopian tubes in patients with endometriosis and or primary or secondary infertility and is a useful diagnostic and therapeutic tool in patients with adenomyosis [35]. In diagnostic hysteroscopy typical findings have been reported in the last years [36]. Molinas and Campo described irregular endometrium with endometrial defects, altered vascularization, and cystic hemorrhagic lesions as possible signs associated with adenomyosis [37]. Superficial openings on the endometrial cavity, hypervascularization, and cystic hemorrhagic lesions were reported as suggestive for adenomyosis by Graziano et al. [22]. However, a pathognomonic feature for adenomyosis in hysteroscopy does not exist. Additionally, hysteroscopy allows the retrieval of targeted endomyometrial biopsies. In transvaginal ultrasound, localization and diameter of the suspicious lesion and its profundity in relation to the thickness of the uterine wall can be described. Thus, a minimally invasive, tissue-sparing biopsy can be obtained as a proof to the disease. In a cross-sectional study with 292 patients clinically suggestive of having adenomyosis, Dakhly et al. investigated the accuracy of endomyometrial biopsy obtained by office hysteroscopy for the histopathologic proof of adenomyosis. Adding hysteroscopic endomyometrial biopsy to the result of TVS improved the specificity from 60 to 89% [24]. Already in 1992 McCausland concluded that hysteroscopic myometrial biopsy can diagnose adenomyosis [50]. The certainty of a positive histopathological result makes it easier to understand the presence of adenomyosis for the patients and facilitates non-evidence-based therapeutic decisions like the use of GnRH analogues or temporary LNG-IUDs in patients with adenomyosis and infertility. In adenomyosis hysteroscopy is not just a diagnostic tool, but also a minimally invasive approach in the treatment of subendometrial and myometrial cystic adenomyomas [51, 52] or polypoid adenomyomas [53] by monopolar or bipolar hysteroscopic resection.

### 3.6. Laparoscopy

In patients undergoing laparoscopy for peritoneal or deep infiltrating adenomyosis, the uterus can be evaluated during surgery and the suspicion of additional adenomyosis can be substantiated with the laparoscopic uterine appearance. Uterine enlargement, a pillowy resistance of the uterine wall, and cystic subserous hemorrhagic lesions are possible laparoscopic parameters in adenomyosis. Another characteristic finding can be the “blue sign,” which describes the color of the adenomyotic tissue during blue dye test [22]. Undirected laparoscopic biopsy techniques without prediagnosed suspicious lesions are not helpful due to unsatisfactory sensitivity and specificity in order to proof adenomyosis histologically. Visible subserous cystic lesions can be resected laparoscopically [38] and focal or diffuse adenomyomas can be treated by laparoscopic adenomyomectomy in order to reduce symptoms [39, 40, 54] and thus biopsies can be obtained.

## 4. Conclusions

A reliable diagnose of adenomyosis can be made by the combination of clinical history, gynecological examination, and transvaginal 2D and 3D ultrasound. In addition, Doppler sonography and MR imaging might help to determine adenomyosis, especially in cases with combined uterine fibroids. Histologic certainty can be achieved by targeted hysteroscopic biopsy following transvaginal ultrasound with localization of the adenomyotic lesions. Thus, the presence or absence of adenomyosis can be included in the individual treatment concept of every patient. More detailed prospective studies are needed in order to determine the accuracy of the different diagnostic tools in adenomyosis.

## Figures and Tables

**Table 1:** Diagnostic methods and their typical findings in adenomyosis.

Diagnostic method	Findings	References
Clinical history and symptoms	Dysmenorrhea, abnormal bleedings, pelvic pain, dyspareunia	[1, 16–19]

Gynecological examination	Uterine and pelvic pain, uterine size and mobility, deep infiltrating endometriosis	[1, 12, 20]

2D transvaginal ultrasound	Heterogeneous myometrium, hyperechoic linear striation in the myometrium, myometrial anechoic cysts, subendometrial microcysts, asymmetrical myometrial thickening, globally uterine enlargement, question mark sign, thickening of the junctional zone, hyperechoic myometrial areas	[21–26]

3D transvaginal ultrasound	JZ (max) > 8 mm, myometrial asymmetry, hypoechoic striation	[26–29]

MR imaging	JZ (max) > 12 mm, high-signal-intensity myometrial spot, JZ (max) to myometrial thickness ratio >40%	[30–34]

Hysteroscopy	Irregular endometrium, endometrial defects, altered vascularization, cystic lesions	[22, 24, 35–37]

Laparoscopy	Uterine enlargement, pillowy resistance, “blue sign,” cystic subserous lesions	[22, 38–40]
